# The Re-Emergence of Neuroinvasive Flaviviruses in Croatia During the 2022 Transmission Season

**DOI:** 10.3390/microorganisms12112210

**Published:** 2024-10-31

**Authors:** Maja Bogdanic, Vladimir Savic, Ana Klobucar, Ljubo Barbic, Dario Sabadi, Morana Tomljenovic, Josip Madic, Zeljka Hruskar, Marcela Curman Posavec, Marija Santini, Vladimir Stevanovic, Suncica Petrinic, Ljiljana Antolasic, Ljiljana Milasincic, Mahmoud Al-Mufleh, Dobrica Roncevic, Tatjana Vilibic-Cavlek

**Affiliations:** 1 Department of Virology, Croatian Institute of Public Health, 10000 Zagreb, Croatia; maja.bogdanic@hzjz.hr (M.B.); zeljka.hruskar@hzjz.hr (Z.H.); ljiljana.antolasic@hzjz.hr (L.A.); ljiljana.milasincic@hzjz.hr (L.M.); 2 School of Medicine, University of Zagreb, 10000 Zagreb, Croatia; marijasantini.ms@gmail.com; 3 Poultry Center, Croatian Veterinary Institute, 10000 Zagreb, Croatia; v_savic@veinst.hr; 4 Department of Epidemiology, Andrija Stampar Teaching Institute of Public Health, 10000 Zagreb, Croatia; ana.klobucar@stampar.hr (A.K.); marcela.curman@stampar.hr (M.C.P.); suncica.petrinic@stampar.hr (S.P.); 5 Department of Microbiology and Infectious Diseases with Clinic, Faculty of Veterinary Medicine, University of Zagreb, 10000 Zagreb, Croatia; ljubo.barbic@vef.hr (L.B.); josip.madic@gmail.com (J.M.); vladostevanovic@gmail.com (V.S.); 6 Department of Infectious Diseases, Clinical Hospital Center Osijek, 31000 Osijek, Croatia; dariocroatia@gmail.com; 7 Medical Faculty, Josip Juraj Strossmayer University of Osijek, 31000 Osijek, Croatia; 8 Department of Epidemiology, Primorje-Gorski Kotar Teaching Institute of Public Health, 51000 Rijeka, Croatia; tomljenovicmorana@gmail.com (M.T.); dobrica.roncevic@zzjzpgz.hr (D.R.); 9 Department of Social Medicine and Epidemiology, Faculty of Medicine, University of Rijeka, 51000 Rijeka, Croatia; 10 Department for Infections in Immunocompromised Patients, University Hospital for Infectious Diseases “Dr. Fran Mihaljevic”, 10000 Zagreb, Croatia; 11 Department of Infectious Diseases, County Hospital Cakovec, 40000 Cakovec, Croatia; mahmoud.almufleh@gmail.com; 12 Department of Public Health, Faculty of Health Studies, University of Rijeka, 51000 Rijeka, Croatia

**Keywords:** tick-borne encephalitis virus, West Nile virus, Usutu virus, epidemiology, Croatia

## Abstract

(Re-)emerging arboviruses, such as tick-borne encephalitis virus (TBEV), West Nile virus (WNV), and Usutu virus (USUV), are continuously increasing in incidence. We analyzed the epidemiological characteristics of flavivirus infections in humans, sentinel animals, and mosquitoes detected in the 2022 transmission season in Croatia. From April to November 2022, 110 hospitalized patients with neuroinvasive diseases (NID) were tested for the presence of arboviruses. RT-qPCR was used to detect TBEV, WNV, and USUV RNA. An ELISA and virus neutralization tests were used for the detection of flavivirus antibodies. TBEV infection was confirmed in 22 patients with NID. WNV NID was detected in six patients. TBE showed male predominance (81.8%; male-to-female ratio of 4.5:1). All but one WNV patients were males. TBE occurred from April to August, with the majority of patients (83.3%) being detected during the May–June–July period. WNV infections were recorded in August and September. In addition to human cases, asymptomatic WNV infections (IgM positive) were reported in 10 horses. For the first time in Croatia, WNV NID was observed in one horse that presented with neurological symptoms. Furthermore, USUV was confirmed in one dead blackbird that presented with neurological symptoms. A total of 1984 mosquitoes were collected in the City of Zagreb. Two *Ae. albopictus* pools tested positive for flavivirus RNA: one collected in July (USUV) and the other collected in August (WNV). A phylogenetic analysis of detected human and avian strains confirmed WNV lineage 2 and the USUV Europe 2 lineage. The presented results confirm the endemic presence of neuroinvasive flaviviruses in continental Croatia. The continuous monitoring of virus circulation in humans, sentinel animals, and mosquitoes is needed to reduce the disease burden.

## 1. Introduction

(Re-)emerging flaviviruses, such as tick-borne encephalitis virus (TBEV), West Nile virus (WNV), and Usutu virus (USUV), represent major public health problems in Europe, with continuous increases in incidence as well as geographic expansion [1,2,3].

TBEV is one of the most significant arboviruses affecting the human central nervous system (CNS). The disease is endemic in 27 Central, Northern, and Eastern European countries, and an increase in morbidity was noted [4]. A gradual increase in TBE cases has been observed in Europe since 2017 with higher notification rates among males and adults aged 45–64 years. Most cases continue to occur from spring to autumn, with no evidence of major changes in seasonal patterns [5]. Besides humans, sporadic TBEV infections were reported in horses and dogs [6,7].

In the past decade, WNV caused remarkable outbreaks in certain areas of Europe. The largest outbreak of human WNV infections in the European Union (EU)/European Economic Area (EEA) countries was recorded in 2018 when the number of infections exceeded the number of all reported infections between 2010 and 2017. In addition, the highest number of newly affected areas was reported during this outbreak [8,9]. Although WNV neuroinvasive disease can occur in any age group, meningitis, encephalitis, and myelitis are more common in elderly individuals and patients with underlying diseases. WNV infections showed a strong seasonal pattern with a peak in August. In addition to human cases, WNV outbreaks among equids and birds were repeatedly recorded in Europe [10].

USUV is still a neglected arbovirus in many countries, although reported human cases are rising. Clinical cases of USUV neuroinvasive infections have been recorded in Italy since 2009 [11,12], France (2016) [13], Hungary (2018) [14], Czech Republic (2018) [15], and more recently in Austria (2021) [16]. The geographic and seasonal distribution of USUV overlaps that of WNV [17]. Epizootics in birds with significant mortality were recorded in Europe in 2016 and 2018 [18,19].

In Croatia, TBEV, WNV, and USUV are detected in continental regions. TBEV is endemic in northwestern Croatian counties. The number of reported human cases ranges from four to forty-five per year with an incidence of 0.14 to 1.05 per 100,000 inhabitants [20]. In recent years, TBEV emerged in some local areas in the mountainous region of Gorski Kotar, which divides the continental area from the coastal area [21,22]. In addition to acute human cases and seropositive individuals, TBEV antibodies were detected in horses, sheep, and goats [22]. TBEV RNA was also found in ticks removed from red foxes (*Vulpes vulpes*) and in deer (*Cervus elaphus*) spleen [23].

Outbreaks as well as sporadic human WNV infections were reported continuously in Croatia from 2012 to 2018 [24]. However, acute asymptomatic (IgM positive) infections and IgG seropositive horses were notified each year, but no clinical WNV infections have been reported so far. WNV infections in birds (two dead goshawks, *Accipiter gentilis*, and one seropositive buzzard, *Butteo butteo*) were detected during the largest Croatian outbreak in 2018 [25]. Serologic evidence of WNV infection was also confirmed in poultry and pet animals (dogs and cats) [26]. So far, there have been no reports of WNV detection in mosquitoes in Croatia.

Human neuroinvasive USUV infections were notified in Croatia during the 2013 and 2018 WNV outbreaks [25]. Two USUV-seropositive horses were confirmed in 2011, while two USUV-positive dead blackbirds (*Turdus merula*) were detected in 2018. USUV antibodies were also found in sheep and one cat [26]. Furthermore, USUV was detected in one mosquito pool from each of the following years: 2016 (*Aedes albopictus*), 2017 (*Culex pipiens* complex), 2018 (*Cx. pipiens* complex), and 2019 (*Cx. pipiens* complex) [27].

A phylogenetic analysis of detected Croatian flavivirus strains in humans, animals, and vectors confirmed the presence of the TBEV European subtype, WNV lineage 2, and USUV Europe 2 lineage [23,26,27].

In 2022, WNV and USUV re-emerged in Croatia after a three-year absence. This study analyze the epidemiological characteristics and molecular epidemiology of flavivirus infections in humans and animals (“One Health”) detected during the 2022 transmission season. Furthermore, we discuss the impact of climate change and mosquito population dynamics as possible drivers of flavivirus re-emergence in Croatia.

## 2. Materials and Methods

**Figure 1:**
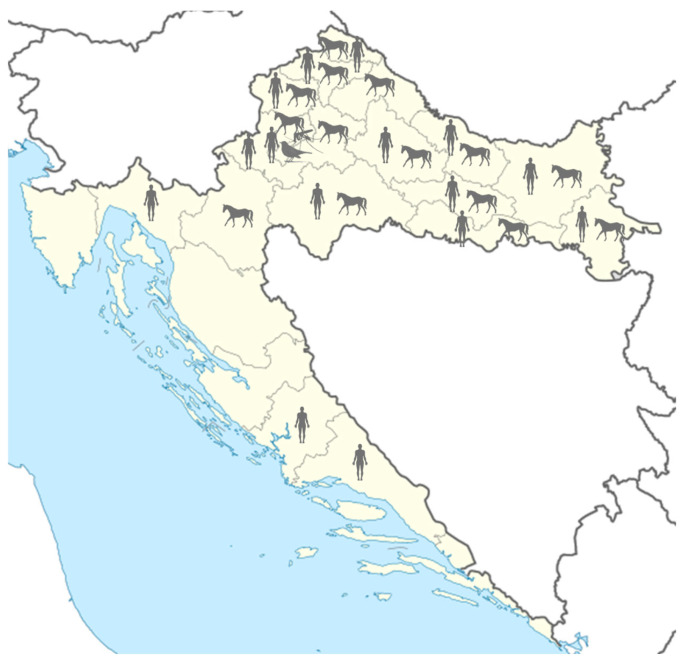
Sampling areas for flavivirus testing in 2022.

From April to November 2022, 110 hospitalized patients with suspected neuroinvasive arboviral infections were tested for TBEV, WNV, and USUV. In addition, a total of 507 horses from continental regions were tested for WNV. One dead blackbird was tested for WNV and USUV. During the entomologic survey, a total of 1984 mosquitoes were collected and identified in the City of Zagreb. Mosquitoes were tested for the presence of WNV and USUV. The sampling area is presented in Figure 1.

We analyzed the epidemiological and clinical characteristics of patients with confirmed acute flavivirus infection (TBEV, *n* = 22; WNV, *n* = 6), horses with WNV (*n* = 11), and a bird with USUV infection.

### 2.1. Serological and Molecular Testing of Human Samples

In humans, the diagnosis was confirmed by the detection of viral RNA in the cerebrospinal fluid (CSF) or urine (WNV) and/or specific IgM antibodies in the CSF (TBEV, WNV). To extract viral RNA, a High Pure Viral Nucleic Acid Kit (Roche Applied Science, Penzberg, Germany) was used. TaqMan real-time reverse-transcription polymerase chain reaction (RT-qPCR) specific assays for the detection of TBEV, WNV, and USUV RNA were performed according to the protocols of Schwaiger and Cassinotti (2003) [28], Tang et al. (2006) [29], and Nikolay et al. (2014) [30], respectively.

TBEV, WNV, and USUV IgM and/or IgG antibodies and IgG avidity were determined using commercial enzyme immunoassays (ELISA; Euroimmun, Lübeck, Germany). The ELISA results were interpreted as follows: IgM ratio < 0.8 as negative, 0.8–1.1 as borderline, and >1.1 as positive; IgG RU/mL < 16 as negative, 16–22 as borderline, and >22 as positive. IgG avidity was tested using urea as a denaturing agent (Avidity ELISA; Euroimmun, Lübeck, Germany). The avidity index (AI) was calculated and interpreted as follows: <40%, low (acute/recent infection); 40–60%, borderline; and >60%, high (previous exposure).

Primers and probes for the RT-PCR and serological tests used for the detection of flaviviruses are presented in Table 1.

Samples with cross-reactive flavivirus antibodies were confirmed using a virus neutralization test. The virus titer (median tissue culture infectious dose; TCID_50_) was calculated using the Reed and Muench formula. Serial two-fold dilutions of heat-inactivated serum samples (30 min/56 °C) starting at a 1:5 ratio were prepared in duplicate in 96-well microtiter plates using Dulbecco’s Modified Eagle Medium (DMEM; Lonza, Basel, Switzerland). A mixture of 25 μL inactivated serum dilutions and 25 μL 100 TCID_50_ of the virus was incubated for 1 h at 37 °C. In the final phase, 50 μL of 2 × 10^5^ Vero E6 cells/mL in DMEM with 10% heat-inactivated fetal calf serum (Capricorn Scientific, Ebsdorfergrund, Germany) were added to each well. Positive and negative controls were included. The plates were incubated at 37 °C with CO_2_ for five days and examined for the cytopathic effect starting on the third day of incubation. Antibody titer ≥ 1:10 was considered positive [31].

### 2.2. Serological and Molecular Testing of Animal Samples

In horses, WNV IgM antibodies were detected in serum samples using a commercial ELISA (INgezim West Nile virus IgM, Gold Standard Diagnostics, Madrid, Spain). Inhibition percentage (IP) was calculated according to the following formula: IP = 100 − [(sample OD/negative control OD) × 100]. Samples with IP ≤ 30% were considered negative, 30–40% equivocal, and ≥40% positive. In a bird, USUV RNA was detected in brain tissue by RT-qPCR as described above [30].

### 2.3. Collection and Molecular Testing of Mosquito Samples

Different traps and methods were used to collect mosquitoes, including CDC Mini Light traps (BioQuip, Products, Rancho Dominguez, CA, USA) and aspirator collection.

CDC Mini Light traps were equipped with dry ice (CO_2_) as an attractant. This type of trap is the most commonly used for WNV surveillance, sampling various species of host-seeking mosquitoes or gravid mosquitoes seeking a place to lay eggs [32]. Traps were placed approximately 1.5 m from the ground and set in the late afternoon before sunset, left overnight, and removed after sunrise (07:00–10:00). During 2022, traps with CO_2_ were set at the same eight collection sites every 14 days from May to October. A total of 88 sampling occasions were gathered. The following habitats were chosen for sampling: the woods (three locations), a populated area close to the green belt (two locations), gardens in the urban part of the city (two locations), and a city center close to the Zagreb botanical garden (one location). During the summer of 2022, mosquitoes were collected using the Human Landing Collection method (HLC) with an aspirator in the settlement Cvjetno Naselje (as a part of the preparation process for a project that uses sterile mosquitoes). Mosquitoes were prepared for identification and analysis with a total of 16 samplings.

The mosquitoes sampled using traps with CO_2_ were transported to the laboratory in containers with dry ice, transferred to plastic tubes, and stored on dry ice until identification. Mosquitoes collected using the aspirator were transported alive to the laboratory in the aspirator, placed briefly in a freezer at −18 °C, and then identified. Female mosquitoes were morphologically identified by species or species complex on a chilling surface under a stereomicroscope using the determination key by Becker et al. (2020) [33]. Specimens belonging to the same species/complex collected on the same day and at the same sampling site were pooled, with up to 60 individuals per pool, and stored at −80 °C until virological testing.

Mosquito pools were tested for WNV and USUV RNA by RT-qPCR as described above [29,30].

### 2.4. Genotyping of Human and Animal Samples

Two strains detected by RT-qPCR (a WNV strain from the urine of a patient with the neuroinvasive disease and USUV from a bird brain) were further subjected to conventional RT-PCR according to the protocol by Weissenbock et al. (2002) [34], which amplifies a wide range of mosquito-borne flaviviruses. Forward (5′-TACAACATGATGGGVAARAGAGAGA-3’) and reverse (5’-AGCATGTCTTCYGTBGTCATCCAYT-3’) primers resulting in a 1084 bp amplification product were used. The RT-PCR amplification products were Sanger sequenced (Humanizing Genomics, Macrogen Inc., Seoul, Republic of Korea) using the same primers. Genotyping and phylogenetic grouping of the obtained sequences were carried out based on a comparison with strains retrieved from the GenBank and obtained using the BLAST algorithm (http://www.ncbi.nlm.nih.gov, accessed on 12 August 2024). A maximum likelihood phylogenetic analysis was conducted, and the evolutionary analyses were performed by using MEGA11 [35].

### 2.5. Statistical Analysis

The differences between groups were compared using the chi-square and Fisher’s exact tests. iCalcu.com Statistic Calculators was used for statistical analysis (available from: https://www.icalcu.com/stat/index.html, accessed on 18 October 2024).

## 3. Results

### 3.1. Human Flavivirus Infections

**Figure 2:**
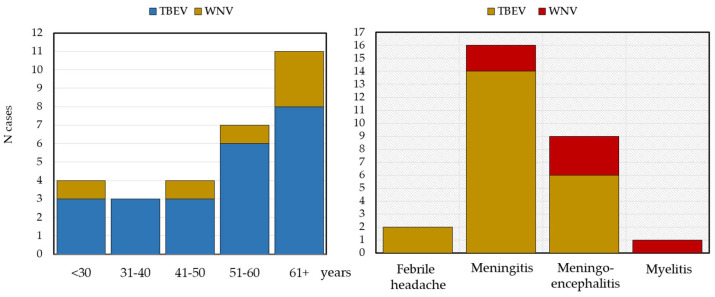
Distribution of patients with flavivirus infections by age (**left**) and clinical presentation (**right**).

TBEV infection was confirmed in 24 patients aged 15–72 years. Twenty-two infections were autochthonous (18 males and 4 females); two were imported from Germany (Bavaria) and Poland. The male-to-female ratio was 4.5:1. The majority of patients were in the age group of 61+ years (8; 34.8%), but infections were recorded in all age groups, with incidence ranging from 13.0 to 26.1% (Figure 2). The median patient age was 58 years (IQR = 39–67). Clinical presentations of TBE included “febrile headache” (*n* = 2), meningitis (*n* = 14), and meningoencephalitis (*n* = 6) (Figure 2). WNV was detected in six patients (five males/one female) aged 13–66 years. The median patient age was 57 (IQR = 44–64) years. The patients with WNV presented with meningitis (*n* = 2), meningoencephalitis (*n* = 3), and myelitis (*n* = 1) (Figure 2).

### 3.2. West Nile Virus Infections in Horses

Acute asymptomatic WNV infections (IgM positive) were recorded in 10 horses from Zagreb, Zagreb County, and Brod-Posavina County. For the first time in Croatia, a clinically overt WNV neuroinvasive infection was confirmed one horse from Osijek-Baranja County, the easternmost part of the country, which is an endemic WNV area.

Case presentation: The clinical symptoms in the horse with no vaccination history started on 15 August with a fever (40.2 °C). The first complaints were loss of appetite and lethargy. After initial treatment with non-steroidal anti-inflammatory drugs, the animal was afebrile after 48 h but developed mild depression and generalized weakness. The animal was referred to the Veterinary Teaching Hospital of the Faculty of Veterinary Medicine at the University of Zagreb for further treatment. Over the next two days, clinical signs progressed to muscle fasciculations, especially in the neck area, followed by generalized muscle tremors that increased during animal handling. Excessive sweating, teeth grinding, and salivation were also observed. The blood samples tested negative for WNV using RT-qPCR. Using an ELISA, WNV IgM antibodies were detected in serum samples. According to the World Organization for Animal Health Terrestrial Animal Health Code, WNV IgM detection in an unvaccinated animal with clinical signs consistent with WNV infection is considered a confirmed WNV case [36]. Five days later, with symptomatic treatment and supportive care, the neurological signs were no longer present, and the horse recovered completely.

### 3.3. Usutu Virus Infection in a Blackbird

USUV was confirmed in a dead blackbird (*Turdus merula*) from Zagreb that presented with neurologic symptoms.

Case presentation: On 26 August, an adult female blackbird was found lethargic, unresponsive, and having seizures. The bird died a few minutes after it was found. Within a flavivirus surveillance program, the bird was sent to the Poultry Center, Croatian Veterinary Institute for flavivirus testing. Using a RT-qPCR, USUV RNA was detected in the brain tissue, confirming USUV infection.

### 3.4. West Nile Virus and Usutu Virus Detection in Mosquitoes

In 2022, a total of 1984 mosquitoes were collected and identified in the City of Zagreb. The mosquitoes belonged to nine species: *Anopheles plumbeus*, *Ae. albopictus*, *Ae*. *geniculatus*, *Ae. rossicus*, *Ae. rusticus*, *Ae. sticticus*, *Ae. vexans*, *Coquillettidia richiardii*, and *Cx. pipiens* complex. Of these, 1366 individuals were tested for the presence of WNV and USUV RNA, namely 59.8% *Ae. albopictus* mosquitoes, followed by *Oc. sticticus* (35.1%), *Cx. pipiens* complex (1.9%), and *Ae. geniculatus* (1.1%). Other species were represented by less than 1% (Table 2). *Aedes vexans* mosquitoes were previously analyzed in the research on Tahyna orthobunyavirus in urban areas in Croatia [37].

The mosquito specimens were sorted in 69 pools and tested for the presence of WNV and USUV (Table 2). One WNV-positive pool and one USUV-positive pool were detected. The WNV-positive mosquitoes were *Ae. albopictus* trapped on July 27. The WNV-positive pool contained 19 mosquitoes, which were collected via human landing collection in a park and adjacent yard in the settlement Cvjetno Naselje in Zagreb. The USUV-positive pool contained 24 *Ae. albopictus* mosquitoes, which were also trapped via human landing collection on 2 August in Cvjetno Naselje in two adjacent yards. Due to the high RT-qPCR cycle threshold (Ct WNV, 32.44; USUV, 33.53), sequencing was not successful.

### 3.5. Seasonal and Geographic Distribution of Flavivirus Infections

**Figure 3:**
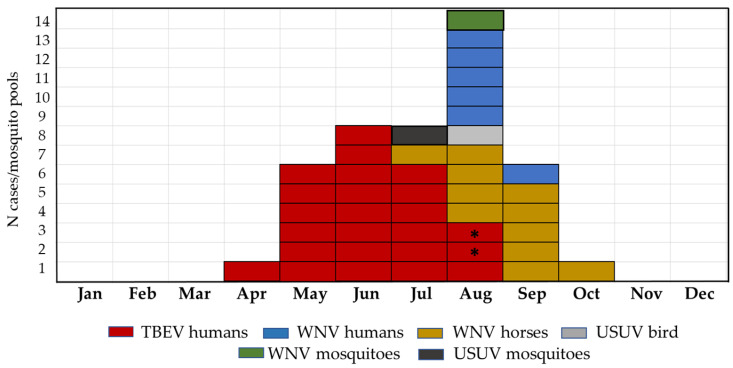
Seasonal distribution of flavivirus infections in 2022 (* represents imported cases).

The seasonal distribution of flavivirus infections detected in the 2022 transmission season (clinical infections in humans, a horse, and a bird as well as asymptomatic acute infections in horses and positive mosquito pools) is presented in Figure 3. TBEV occurred from April to August, with the majority of patients being detected during the May–June–July period (20; 83.3%). Human WNV infections were recorded in August (5; 83.3%) and September. WNV infections in horses were reported from July to October, peaking in August (4; 36.4%) and September (5; 45.5%). The USUV-positive bird was found in August. Positive mosquito pools were detected in July (USUV) and August (WNV).

**Figure 4:**
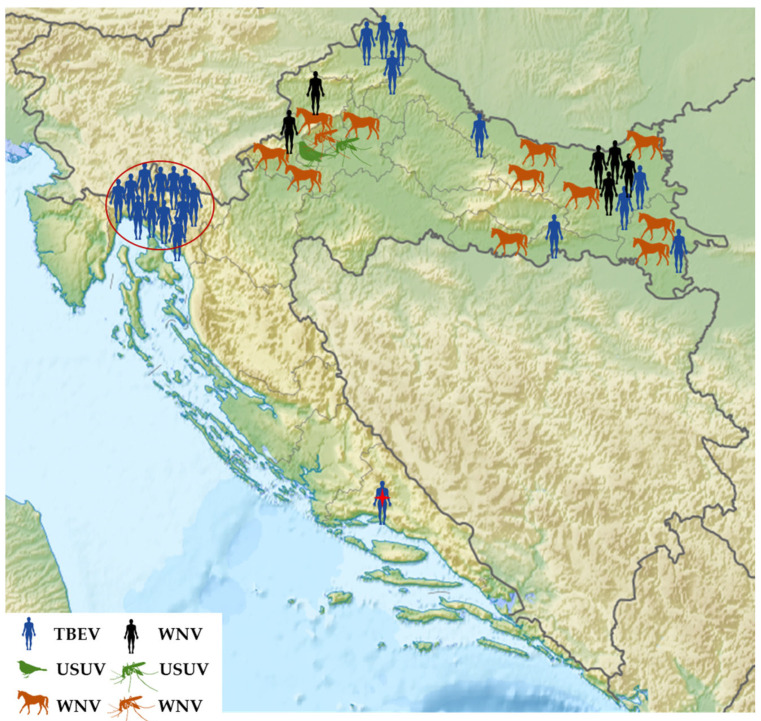
The geographic distribution of flavivirus infections in 2022. The TBEV-positive patient from a coastal region labeled with a red asterisk was exposed to the virus in the Gorski Kotar area (circled in red).

In addition to sporadic infections in six continental regions, a cluster of 13 TBEV cases was detected in Gorski Kotar, a mountainous area between continental and coastal Croatia. One patient with TBE from Split-Dalmatia County stayed in Gorski Kotar. Human WNV infections were recorded in four continental counties. WNV infections in horses were observed in seven continental counties, while the USUV-positive blackbird was detected in Zagreb city (Figure 4).

### 3.6. Molecular Epidemiology of Flavivirus Infections

**Figure 5:**
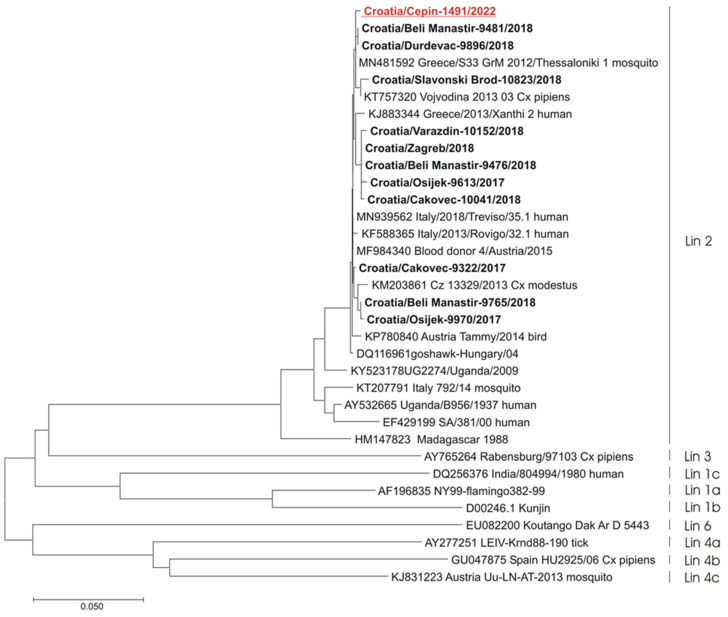
A phylogenetic tree of West Nile virus. The evolutionary history was inferred on 848 positions of the NS5 gene. The scale bar indicates nucleotide substitutions per site. WNV isolates from humans in Croatia are marked in bold. In addition, the isolate from 2022 is underlined and shown in red. The WNV lineages proposed by Rizzoli et al. (2015) [38] are denoted on the right.

**Figure 6:**
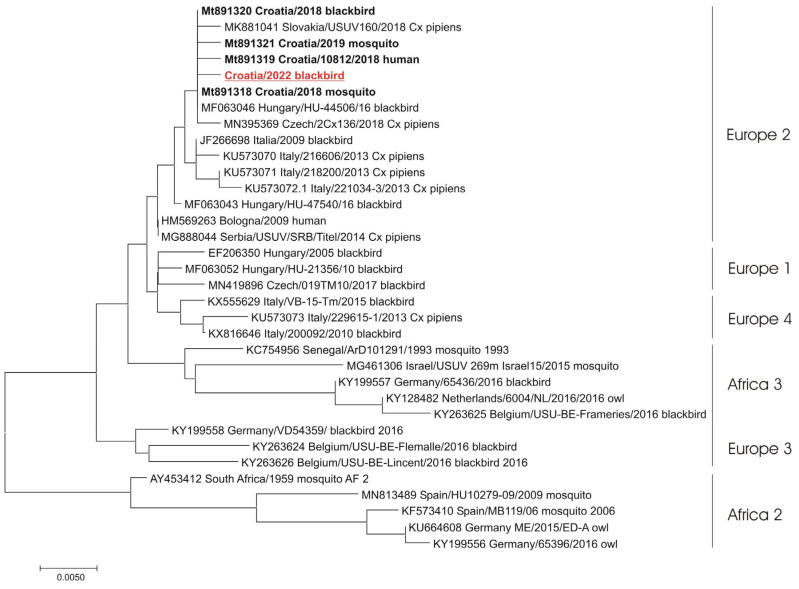
The evolutionary history was inferred on 543 positions of the NS5 gene. The scale bar indicates nucleotide substitutions per site. The USUV isolates from Croatia are marked in bold. In addition, the isolate from 2022 is underlined and shown in red. The USUV lineages proposed by Cadar et al. (2017) [39] are denoted on the right.

A phylogenetic analysis of detected flavivirus strains showed the presence of WNV lineage 2 (one human case) (Figure 5) and the USUV Europe 2 lineage (blackbird) (Figure 6).

### 3.7. Prevalence of West Nile Virus Infections According to Species and Geographic Region

The prevalence of acute WNV infections according to species and geographic region is presented in Table 3 and Table 4. No significant differences were observed between species (*p* = 0.120) or regions (humans, *p* = 0.304; horses, *p* = 1.000).

## 4. Discussion

In continental Croatia, human clinical TBE cases were continuously detected with the highest incidence in the northwestern and eastern counties [22]. In 2022, 22 autochthonous cases were detected, while 2 infections were imported from Germany and Poland. When analyzing the seasonal distribution of patients with TBE in Croatia, in 2022, cases were reported from April to August with a peak in June (36.4%). In previous years, bimodal TBE seasonal activity was dominant in Croatia with a larger peak in the May–July period and a smaller one in the October–November period [20]. In the rest of Europe, the epidemiology of TBE is characterized by annual variations with an increasing trend. A year-round TBE transmission was observed, with 98.8% of cases occurring between April and November. The distribution of autochthonous cases was bimodal in every year but 2012 and 2016, with the first peak occurring around the first week of July and a second peak occurring at the end of September [40].

Like in our study (male-to-female ratio of 4.5:1), the male sex predominated in TBE in Europe (1.5:1) [40]. When analyzing the age of hospitalized patients with TBE in Croatia, the median age was 58 years, with the majority of patients (36.4%) being older than 60 years. The same age group was identified as the group with the highest number of infections (33.3%) during previous transmission seasons [22]. Data from Europe show that the median ages of patients with TBE were 49 years (unvaccinated individuals) and 57 years (vaccinated individuals) [40].

The main clinical presentation in Croatian patients was meningitis, which was recorded in 63.6% of patients. Similar clinical presentations were observed in Germany (meningitis, 31.5%; encephalitis or myelitis, 21.5%) [41]. In the European multicenter study conducted from 2010 to 2017, meningitis and meningoencephalitis were detected in 37.3% and 49.2% of patients, respectively [42]. In addition, meningoencephalitis was the dominant clinical form in Lithuania (82%) [43].

After 2018, an unusually intense WNV transmission season was documented in Europe in 2022 with 1133 confirmed and probable human WNV cases reported [44]. Similarly, human WNV cases re-emerged in Croatia after a three-year absence since the largest outbreak so far in 2018 [25]. Comparing 2022 with 2018, Italy experienced a very intense season. By late August, most cases (76%) and deaths caused by WNV infections (71%) in the EU/EEA were reported in Italy. In July, there was an even higher number of human WNND cases compared to the same period in 2018 [45]. A quite intense WNV season in 2022 was also observed in Greece. It was the third most intense season (286 diagnosed human WNV infections with 184 presenting with WNND) following 2018 and 2010. At the municipality level, in three new municipalities, human WNV infections were recorded for the first time [46,47].

In Croatia, the seasonal occurrence of WNV in 2022 (August–September) was similar to previous years, while in horses, acute infections were registered from July to October. In contrast, the 2022 transmission season in Italy was characterized by an early WNV circulation in mosquitoes and birds, but human infections were limited until early July [45]. The first WNV-positive *Culex* mosquito pool was detected in early June in the Veneto region. Afterward, two blood donors tested positive, as well as patients with encephalitis, wild birds, and additional mosquito pools. The early start of the WNV season may have resulted from changes in weather conditions, with the period from March to May in 2022 being exceptionally dry and hot in Northern Italy [48]. A nationwide average increase of roughly 2 and 2.7 °C in temperature in May and June, respectively, compared with the mean of the previous ten years, was probably the driver of increased WNV transmissibility in 2022. Concurrently, there was a trend toward drier weather, with average relative humidity drops of 3.9 and 6.5% in May and June 2022, respectively. This corroborates the hypothesis that the early WNV transmission season in Italy in 2022 is related to unusual weather [49]. A similar early start of the season was observed in Greece, with the first human case developing symptoms in June [46]. Similarly to Italy, the earlier start and intense 2022 WNV circulation may have been influenced by meteorological conditions (a warm and dry spring and a warm and extremely rainy early summer with local floods, notably in the north and central mainland around the epicenters) as was also proposed for 2018 [46]. An association of WNV infections with air temperature was observed during the 2018 outbreak in Croatia as well [25].

An artificial intelligence-based model identified high spring/summer temperatures as the main determinants of WNV outbreaks in Europe. The most important driver of the WNV outbreaks throughout Europe was the mean temperature of the warmest quarter of the preceding year. In areas where WNV was present, the mean temperature of the warmest quarter varied from 20 to 26 °C. Similarly, a higher-than-usual spring temperature in this range (22–26 °C) early in the year may also signal the start of a WNV outbreak in the second half of the same year [50]. The temperature was one major factor contributing to the huge WNV epidemics throughout Europe in 2018 [48].

The extent to which climate change contributes to the spatial expansion of WNV in Europe was examined in a recently published study, which also took into consideration other direct human factors including land use and population changes. Although anomalous higher spring temperatures have been shown to be a reliable early warning indicator for human WNV cases, it has been suggested that the relative contributions of summer and winter air temperatures to the ecological suitability of WNV may be greater than those of spring temperatures [51].

Climate changes may affect the vector distribution and dynamics. The principal vectors of WNV exhibit distinct seasonality in different climates. Moreover, viral strains may evolve to varying degrees of pathogenicity in various species and natural situations [52]. In addition to meteorological factors, the risk of mosquito-borne diseases is increased by mosquito species’ adaptation to domestic and urban environments, the abundance of larval habitats in anthropogenic landscapes, and urban microclimates that support mosquito development [53].

Climatic conditions in Croatia, i.e., changes in the precipitation amount in 2022, significantly impacted the total number and dynamics of mosquito populations. The total annual precipitation in 2022 was 798.8 mm, with the highest values being recorded in September (235 mm), November (93.6), and December (107.1) [54]. From January to August, in all months except April, the amount of precipitation was significantly lower than the value of the multi-year average monthly amount of precipitation. In 2022, a total of three times fewer mosquito individuals (1984) were sampled than in 2021, when a total of 5057 were recorded during the sampling process conducted in the same period and at the same localities [55]. In 2022, 9 mosquito species were recorded, while in the previous year, 16 mosquito species were detected. In 2021, the total annual amount of precipitation was approximately the same as in 2022 (772.2 mm), but the precipitation trend during the year was similar to the multi-year average. The highest monthly amount of precipitation was recorded in May (124 mm), which had a significant impact on the high number of flooded mosquito species. Due to the mentioned precipitation trend, the year 2022 is characterized as a dry year. As a result, the dominant species in the entire mosquito sample was the invasive *Ae. albopictus*, also referred to as an urban mosquito breeding species. The flooding mosquito species, *Ae. sticticus* and *Ae. vexans*, are listed below. In contrast, in 2021 *Ae. albopictus* participated with a share of only 12% of the total sampled mosquitoes; flooded species dominated [55].

In 2022, both rural and urban areas in Greece, including large cities, were affected by WNV, unlike previous outbreaks in these regions, which mostly occurred in rural areas [46]. In the United Kingdom, a trend toward an increased risk of WNV around large metropolitan areas characterized by urban heat islands was observed [56]. In our case series, three patients with WNV were from urban areas and three were from suburban/rural areas.

The median age of the Croatian WNV cases was 57 years, which was lower than in Italy (median 70 years) [45] and Greece (76 years) [46]. All but one patient in Croatia were males. In Greece, 58% of the total WNV cases were males [46], while In Italy, the male-to-female ratio was 2:1 [45].

In this study, two patients with WNV presented with meningitis, three with encephalitis, and one with myelitis. A study conducted in the neighboring country Serbia in 2022 showed encephalitis in 84.6% of patients with WNV NID compared with other neurological manifestations such as meningitis and cerebellitis [57].

In addition to human cases, nine Member States reported 364 animal WNV outbreaks to the European Union Animal Diseases Information System (ADIS) in 2022, with 323 avian outbreaks being the highest number reported since 2018 [44]. In addition, nine EU/EEA countries reported 101 outbreaks among equids. In Croatia, asymptomatic acute WNV infections were confirmed in horses as well. Asymptomatic acute infections in horses have been recorded continuously in Croatia since 2012 [24,25]; however, in 2022, a clinically manifest WNV infection was confirmed in one horse for the first time.

Sporadic human USUV infections were also recorded in 2022. In Italy (Reggio Emilia Province), USUV neuroinvasive infection was confirmed in an immunocompromised patient [58]. In addition, USUV was detected in asymptomatic patients during routine examinations before blood donation. This patient represents the first documented case in Asti, Piedmont region (Italy). A total of six patients were reported by the end of the transmission season [12]. In Croatia, no human USUV infections were recorded in 2022; however, USUV RNA was detected in one dead blackbird from the Zagreb area.

Like in previous seasons in Croatia, WNV lineage 2 and the USUV Europe 2 lineage were confirmed. While only WNV lineage 2 circulation was recorded in 2018, the co-circulation of WNV lineages 1 and 2 was observed in Italy in 2022 [45]. The USUV strain detected in a blackbird in the Zagreb area in 2022 clustered within the Europe 2 lineage together with previous Croatian USUV strains detected in a human (2018), a blackbird (2018), and *Cx. pipiens* mosquitoes (2018 and 2019).

For the first time in Croatia, WNV was detected in one *Ae. albopictus* mosquito pool. In addition, one *Ae. albopictus* pool tested positive for USUV by RT-qPCR. However, sequencing was not successful due to the low level of viral RNA. Several studies from different geographic regions, including Croatia, have found WNV and USUV RNA in both *Cx. pipiens* and *Ae. albopictus* mosquitoes. There are several possible explanations for the low levels of WNV and USUV RNA detected in *Ae. albopictus* pools in our study. *Culex pipiens* mosquitoes are found to be more susceptible than *Ae. albopictus* to WNV at lower virus titers [59]. Furthermore, a study from Italy showed a low vector competence of *Ae. albopictus* in transmitting USUV since no evidence of RT-PCR positivity was found in the saliva despite the positive body sample suggesting that a replication in the mosquito body may occur [60]. A very recently published study from France found that *Ae*. *albopictus* can transmit WNV and USUV at 28 °C but not at 20 °C [61].

Based on estimations, the risk of WNV is predicted to increase five times in Europe between 2040 and 2060 compared to the 2000–2020 period depending on the location and climate scenario [50]. In addition, the zoonotic risk associated with USUV avian epizootics in Europe deserves attention even if, to date, human cases remain rare [18]. Therefore, continuous flavivirus surveillance within the “One Health” context is needed to decrease the risk to human health.

There are some limitations of this study that need to be addressed. The main limitation of this study is the small numbers of positive patients and animals. In addition, the majority of patients were from continental Croatian regions where flavivirus infections were recorded in previous years, while mosquito sampling was conducted in a limited area of northwest Croatia. Therefore, the actual number of positive humans, animals, and mosquitoes may be underreported. Since the majority of WNV infections are asymptomatic or present as a mild disease, WNV infections in both humans and horses are also underestimated. Due to the mentioned limitations, the results should not be generalized to other regions in Croatia or Europe.

## 5. Conclusions and Recommendations

Like in some other European countries (intense WNV circulation), human WNV infections reemerged in Croatia in 2022 after a three-year absence. Similarly, USUV was confirmed in one dead blackbird, but no human infections were recorded. USUV and WNV were detected in one *Ae. albopictus* mosquito pool each. The positive WNV pool represents the first detection of WNV in mosquitoes in Croatia. Like in previous transmission seasons, WNV lineage 2 and the USUV Europe 2 lineage were confirmed. After evidence of WNV or USUV infection, mosquito control measures should be implemented. They consist of an integrated approach to mosquito control in the disease-affected area, including the following: the door-to-door inspection of yards and gardens to identify and eliminate mosquito breeding sites while educating citizens; the application of larvicide measures in all natural breeding sites and those that cannot be removed, such as street drains; the sampling of adult mosquitoes for virus analysis; and the application of adulticide measures. In addition, public education campaigns and the improved continuous surveillance of flaviviruses in humans, animals, and vectors (“One Health”) are needed to reduce the disease burden.

## Figures and Tables

**Table 1:** Tests used for the diagnosis of flavivirus infections.

Virus	RT-qPCR	Serology	
	(Humans, Bird, Mosquitoes)	Humans	Horses
TBEV	FP: GGG CGG TTC TTG TTC TCC RP: ACA CAT CAC CTC CTT GTC AGA CT Probe: FAM-TGA GCC ACC ATC ACC CAG ACA CA-TAMRA	IgM/IgG ELISA; IgG Avidity (Euroimmun, Lübeck, Germany)	NT
WNV	FP: AAG TTG AGT AGA CGG TGC TG RP: AGA CGG TTC TGA GGG CTT AC Probe: FAM-CAA CCC CAG GAG GAC TGG-TAMRA	IgM/IgG ELISA; IgG Avidity (Euroimmun, Lübeck, Germany)	IgM ELISA (Ingezim, Gold Standard Diagnostics, Madrid, Spain)
USUV	FP: CAA AGC TGG ACA GAC ATC CCT-TAC RP: CGT AGA TGT TTT CAG CCC ACGT Probe: FAM-AAG ACA TAT GGT GTG GAA GCC TGA TAG GCA-TAMRA	IgG ELISA (Euroimmun, Lübeck, Germany)	NT

TBEV = tick-borne encephalitis virus; WNV = West Nile virus; USUV = Usutu virus; FP = forward primer; RP = reverse primer; NT = not tested.

**Table 2:** The numbers of mosquitoes by species and the sampling methods saved in mosquito pools to test for the presence of West Nile virus and Usutu virus RNA.

Mosquito Species	Sampling Method		Total (%)	Number of Mosquito Pools
	Trap with CO_2_	Aspirator		
*Anopheles plumbeus*	13		13 (0.9)	2
*Aedes albopictus*	509	308	817 (59.8)	43 *^†^
*Aedes geniculatus*	15		15 (1.1)	2
*Aedes rossicus*	1		1 (0.1)	1
*Aedes rusticus*	7		7 (0.5)	2
*Aedes sticticus*	447	33	480 (35.1)	15
*Coquillettidia richiardii*	6		6 (0.4)	1
*Culex pipiens complex*	27		27 (1.9)	3
Total	1025	341	1366	69

* Single pool tested positive for the presence of WNV RNA. **^†^** Single pool tested positive for the presence of USUV RNA.

**Table 3:** Prevalence of West Nile virus infections by species.

Species	N Tested	N (%) WNV Positive	*p*
Humans	110	6 (5.4)	0.120
Horses	507	11 (2.2)	
Mosquitoes	69 *	1 (1.4)	

* Mosquito pools.

**Table 4:** Prevalence of West Nile virus infections by region.

Species	Eastern Continental		Northwestern Continental		Coastal		*p*
	N Tested	N (%) Positive	N Tested	N (%) Positive	N Tested	N (%) Positive	
Humans	42	4 (9.5)	46	2 (4.3)	22	0 (0)	0.304
Horses	264	6 (2.3)	243	5 (2.1)	0	0 (0)	1.000
Mosquitoes	NT	NA	69 *	1 (1.4)	NT	NA	NA

* Mosquito pools; NT = not tested; NA = not applicable.

## Data Availability

The original contributions presented in the study are included in the article, further inquiries can be directed to the corresponding author.
